# Induction of Autophagy and Its Role in Peripheral Nerve Regeneration after Peripheral Nerve Injury

**DOI:** 10.3390/ijms242216219

**Published:** 2023-11-11

**Authors:** Dong Keon Yon, Yong Jun Kim, Dong Choon Park, Su Young Jung, Sung Soo Kim, Joon Hyung Yeo, Jeongmin Lee, Jae Min Lee, Seung Geun Yeo

**Affiliations:** 1 Center for Digital Health, Medical Science Research Institute, Kyung Hee University School of Medicine, Kyung Hee University Medical Center, Seoul 02447, Republic of Korea; yondg@khu.ac.kr; 2 Department of Pediatrics, Kyung Hee University School of Medicine, Kyung Hee University Medical Center, Seoul 02447, Republic of Korea; 3 Department of Pathology, College of Medicine, Kyung Hee University, Seoul 02447, Republic of Korea; yjkim1@khu.ac.kr; 4 Department of Obstetrics and Gynecology, St. Vincent’s Hospital, The Catholic University of Korea, Suwon 16247, Republic of Korea; park.dongchoon@gmail.com; 5 Department of Otorhinolaryngology-Head and Neck Surgery, Myongji Hospital, Hanyang University College of Medicine, Goyang 04763, Republic of Korea; monkiwh35@hanmail.net; 6 Department of Biochemistry and Molecular Biology, College of Medicine, Kyung Hee University, Seoul 02447, Republic of Korea; sgskim@khu.ac.kr; 7 Public Health Center, Danyang-gun, Seoul 27010, Republic of Korea; joonhyungyeo@gmail.com; 8 Department of Otorhinolaryngology–Head and Neck Surgery, College of Medicine, Kyung Hee University Medical Center, Kyung Hee University, Seoul 02453, Republic of Korea; sallyljm@khu.ac.kr (J.L.); sunjaesa@hanmail.net (J.M.L.)

**Keywords:** facial nerve, degeneration, regeneration, autophagy

## Abstract

No matter what treatment is used after nerve transection, a complete cure is impossible, so basic and clinical research is underway to find a cure. As part of this research, autophagy is being investigated for its role in nerve regeneration. Here, we review the existing literature regarding the involvement and significance of autophagy in peripheral nerve injury and regeneration. A comprehensive literature review was conducted to assess the induction and role of autophagy in peripheral nerve injury and subsequent regeneration. Studies were included if they were prospective or retrospective investigations of autophagy and facial or peripheral nerves. Articles not mentioning autophagy or the facial or peripheral nerves, review articles, off-topic articles, and those not written in English were excluded. A total of 14 peripheral nerve studies that met these criteria, including 11 involving sciatic nerves, 2 involving facial nerves, and 1 involving the inferior alveolar nerve, were included in this review. Studies conducted on rats and mice have demonstrated activation of autophagy and expression of related factors in peripheral nerves with or without stimulation of autophagy-inducing factors such as rapamycin, curcumin, three-dimensional melatonin nerve scaffolds, CXCL12, resveratrol, nerve growth factor, lentinan, adipose-derived stem cells and melatonin, basic fibroblast growth factor, and epothilone B. Among the most studied of these factors in relation to degeneration and regeneration of facial and sciatic nerves are LC3II/I, PI3K, mTOR, Beclin-1, ATG3, ATG5, ATG7, ATG9, and ATG12. This analysis indicates that autophagy is involved in the process of nerve regeneration following facial and sciatic nerve damage. Inadequate autophagy induction or failure of autophagy responses can result in regeneration issues after peripheral nerve damage. Animal studies suggest that autophagy plays an important role in peripheral nerve degeneration and regeneration.

## 1. Introduction

### 1.1. Definition and Types of Autophagy

The term ‘autophagy’ is derived from the Greek words ‘auto’ meaning self and ‘phagy’ meaning to eat. There are three types of autophagy: (1) microautophagy, in which intracellular substances directly enter the lysosome through invagination of the lysosomal membrane; (2) macroautophagy, where an autophagosome composed of a double lipid membrane surrounds a substance and fuses with lysosomes; and (3) chaperone-mediated autophagy, which degrades proteins with specific target motifs through a process mediated by a chaperone complex and lysosomal-associated membrane protein type 2A. Where not otherwise specified, ‘autophagy’ generally refers to macroautophagy. Autophagy is further classified into aggrephagy, lipophagy, mitophagy, plexophagy, ribophagy, and xenophagy, depending on the nature of the material that is loaded and digested. Autophagy plays an important role not only in the degradation of protein aggregates, but also in the removal of damaged intracellular organelles including mitochondria, endoplasmic reticulum, and peroxisomes; it also is involved in removing extracellular pathogens, such as bacteria, viruses, and parasites. In various organisms, including mice, whole-body or tissue-specific deletion of autophagy-related genes (Atg) causes serious disorders and death [1,2], supporting the hypothesis that autophagy is an important process in maintaining health.

### 1.2. Molecular Mechanisms Controlling Autophagy

Since the first identification of autophagy-related genes in yeast by Ohsumi et al. in 1993, more than 35 Atg genes have been discovered [3,4]. In addition to being intricately regulated by the proteins it produces, the autophagy process is under negative regulation by mTOR (mammalian target of rapamycin) and positive regulation by AMPK (adenosine monophosphate-activated protein kinase). The process itself can be divided into four major steps: (1) initiation and vesicle nucleation, (2) vesicle elongation, (3) fusion and degradation, and (4) termination. The initiation step is regulated by the ULK1 protein, with the ULK1-containing complex, ULK1-Atg13-Atg101-FIP200, dissociating from the mTORC1 complex as a result of nutrient deficiency-dependent dephosphorylation of ULK1. This leads to increased activation of the beclin-1/Vps34 complex (beclin-1-Atg14L-Vps15-Vps34 complex), inducing the production of PI3P (phosphatidylinositol-3-phosphate) and initiating nucleation of vesicles with a double-membrane structure, facilitated by the DFCP1 and WIPI proteins that gather at the site. Vesicles subsequently elongate through the actions of proteins that constitute the two ubiquitin-like conjugation systems, ATG12-ATG5-ATG16 and LC3-phosphatidylethanolamine, resulting in the formation of an autophagosome. The resulting mature autophagosome then fuses with a lysosome to form an autolysosome, within which isolated intracellular substances or organelles are degraded by lysosomal hydrolytic enzymes. As a consequence, nutrients (e.g., amino acids) released through autolysosomal digestion increase the activation of mTOR protein, a major negative regulator of the mTORC1 complex, ultimately leading to termination of the autophagy process [5,6].

## 2. Induction of Autophagy in Various Diseases

The autophagy process, involving the transport of intracellular substances surrounded by a double membrane structure to a lysosome and subsequent autolysomal degradation, acts together with the ubiquitin proteasome system to play an important role in maintaining intracellular protein quality control. Autophagy also plays an important role in the degradation of protein aggregates and the removal of damaged intracellular organelles and extracellular pathogens. Thus, not surprisingly, dysregulation of autophagy can act as a major cause of various diseases. In the absence of sufficient nutrients to generate adequate ATP, autophagy also breaks down proteins into amino acids, lipid droplets into fatty acids, and glycogen into glucose, thereby serving a regulatory function that maintains intracellular energy balance. Conversely, under energy-replete conditions, autophagic degradation of energy sources (proteins, fat particles, and sugar sources) is suppressed and their storage increases. However, disruptions in the regulation of autophagy result in an energy imbalance [7,8]. 

Ongoing studies have investigated potential positive or negative effects of autophagy on human diseases, but the underlying mechanisms have proven more complex than expected. Consequently, whether and how autophagy is involved in maintaining human health or the development of disease remains unclear. Activation of autophagy in cancer acts as a tumor suppressor, whereas inactivation of autophagy allows the survival of cancer cells under nutrient-poor conditions. In muscular disorders, autophagy may increase as a compensatory response to defects in lysosome function, whereas inactivation of autophagy can lead to the accumulation of autophagosomes, potentially impairing cell function. In neurodegeneration, autophagy activation contributes to the timely removal of protein aggregates before they become toxic, whereas autophagy inactivation might trigger cell death in neurons burdened with aggregated proteins. In the case of pathogen infection, autophagy activation serves as a cellular defense against the invasion of bacteria and viruses; conversely, inactivation of autophagy allows pathogens to establish a replicative niche [7,8,9,10].

The pathological hallmarks of neurodegenerative diseases are associated with the aggregation of misfolded proteins and the loss of specific neuronal populations. Accordingly, autophagy, a major intracellular mechanism in which aggregated proteins and damaged organelles are degraded, has been reported to be involved in pathological changes in many neurodegenerative diseases, including Alzheimer’s disease, Parkinson’s disease (PD), Huntington’s disease (HD), and amyotrophic lateral sclerosis (ALS) [11]. Under normal circumstances, autophagosome vesicles do not exist in the brain. However, dystrophic neurites in Alzheimer’s disease brains contain autophagosomal vesicles, and increased autophagic vacuoles are found in Presenilin 1 (PS1)-rich locations [12]. PD is the second most common neurodegenerative disease. Dysfunctional lysosomes and accumulation of autophagosomes were observed in neurons of postmortem brain samples from PD patients, indicating a pathogenic role of autophagy in PD [13]. The major components of Lewy bodies are misfolded and aggregated *a*-synuclein [14]. *a*-Synuclein levels increase when lysosomes are inhibited, suggesting a close link between α-synuclein degradation and autophagy. HD is a devastating autosomal dominant neurodegenerative disease. Although autophagosome formation is not affected by HD pathology, aggregated autophagosomes are observed in HD models [15]. Huntingtin plays an important role in autophagosome transport. In HD models, depletion of huntingtin leads to abnormal accumulation of autophagosomes with engulfed mitochondria, indicating impaired cargo degradation [16]. Additionally, there is an unusual interaction between autophagy and HD pathogenesis. One polymorphism in Atg7 is known to be associated with an earlier onset form of HD. Another neurodegenerative disease that has been reported to be associated with autophagy is ALS, a fatal paralyzing disease that causes selective loss of motor neurons in the brain and spinal cord, causing muscle weakness and atrophy. Experiments with transgenic mice carrying mutant SOD1 G93A showed that autophagy was activated [17]. Autophagosome aggregation in the cytoplasm indicated that autophagy is activated in degenerating motor neurons of ALS patients. In particular, excessive autophagosomes and autolysosomes are closely associated with p62/SQSTM1-positive inclusions, suggesting impaired cargo digestion in lysosomes. Other studies have shown that increased autophagosome levels are closely associated with decreased phosphorylation of mTOR in a number of genetic ALS models [18].

**Figure 1:**
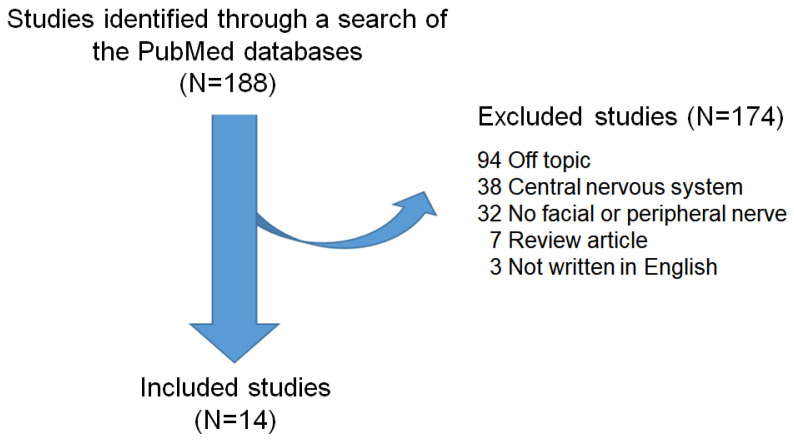
Review flow diagram.

While the involvement of autophagy has been studied in various diseases, its induction and contribution to nerve degeneration and regeneration after facial nerve injury (FNI) has not yet been established. To bridge this knowledge gap and shed light on the clearance of damaged nerve debris and the innate immune response induced by facial nerve damage, we conducted a review of the relevant literature. To this end, we analyzed and summarized the results of previous studies on the involvement of autophagy in nerve regeneration. Our search encompassed literature databases, focusing on studies published in English. Studies were included if they (1) were prospective or retrospective investigations of autophagy and facial or peripheral nerves; (2) considered facial nerve degeneration and regeneration; and (3) included human patients and/or animal studies on autophagy and facial or peripheral nerves. From 1993 to 2023, we identified 188 studies based on search terms from the PubMed electronic database. Articles which did not discuss autophagy or facial or peripheral nerves, review articles, off-topic articles, and articles not written in English were excluded. A total of 14 peripheral nerve studies that met these criteria, including 11 involving sciatic nerves, 2 involving facial nerves, and 1 involving the inferior alveolar nerve, were included in this review (Figure 1).

## 3. Expression of Autophagy-Related Factors, with or without Autophagy Inducers, after Peripheral Nerve Injury

**Figure 2:**
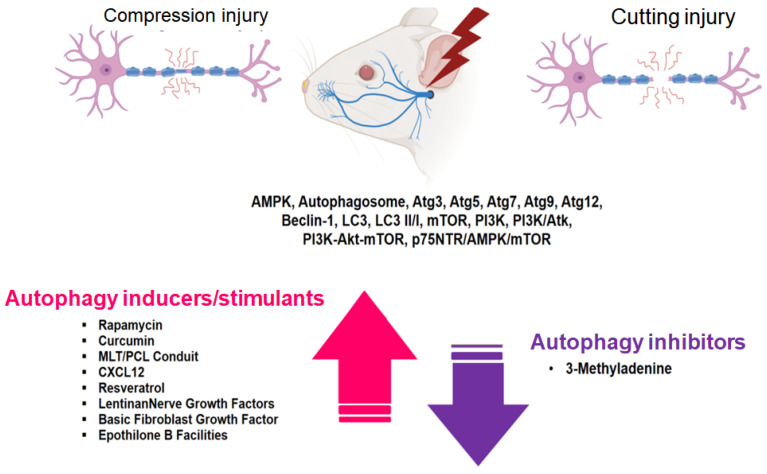
Expression of target substances associated with autophagy in the context of facial and sciatic nerve degeneration and regeneration after nerve injury.

In explorations of the potential role of autophagy in the context of peripheral nerve injury, the expression of autophagy-related factors and associated pathways looms large. This section delves into dynamic changes in these factors and processes in the context of peripheral nerve injury, with or without stimulation of autophagy-inducing factors, particularly as it relates to nerve degeneration/regeneration and recovery (Figure 2).

### 3.1. Upregulation of Autophagy-Related Factors without Stimulation of Autophagy-Inducing Factors after Sciatic Nerve Injury (Table 1) (Figure 3)

One previous study on nerve degeneration and regeneration following sciatic nerve injury in genetically engineered mouse models investigated the modulation of autophagy-related factors after injury [19], reporting distinct upregulation of these factors post-nerve injury. In this study, using Atg7fl/fl, Mpz-Cre, Mpz-Cre, and Mpz-Cre+, in Atg7loxP/loxP mice, the sciatic nerve was exposed and transected, and a temporal analysis of autophagy-related genes and proteins was conducted. During demyelination, several autophagy-related genes, especially those for the ULK complex, ATG9 cycling system, and ATG7, which are essential for autophagosome formation, were strongly induced. Several ATG proteins, including ATG7, ATG16L complex, Wipi2, and beclin-1 (also known as ATG6), were also increased in injured nerves compared with uninjured nerves, concomitant with a decrease in the degeneration of myelin protein zero (MPZ) and myelin basic protein (MBP). Furthermore, a substantial increase in the level of LC3 II, indicative of autophagosome initiation, was observed in response to injury in vivo. An examination of nerve homogenates from uninjured and injured Atg7-cKO mice revealed a significant reduction in ATG7, ATG5-12, and LC3 II in intact and severed nerves, suggesting impaired autophagy. These findings underscore the important role of inductive autophagy in Wallerian degeneration.

Given its role in cellular self-cleaning and repair mechanisms, ATG5 (autophagy-related gene 5), a key player in the autophagy process, holds particular significance in the context of nerve regeneration. In one study investigating whether autophagy induction is important for motor nerve regeneration [20], researchers subjected 12-week-old Sprague–Dawley rats to a nerve crush injury (three times for 30 s with fine forceps) or cutting injury of the right sciatic nerve. After surgery, ATG5 (AAVrh10-ATG5) or control protein, in this case green fluorescence protein (GFP; AAVrh10-GFP), was injected into spinal motor neurons and nerve conduction tests were performed. At 48 and 60 days after nerve injury, rats in the AAVrh10-ATG5 group showed higher nerve conduction amplitudes in gastrocnemius and tibialis anterior muscles than those in the AAVrh10-GFP group (*p* < 0.05). Motor performance of hindlimb movements was also better in the AAVrh10-ATG5 group than in the AAVrh10-GFP group. Collectively, these findings indicate that overexpression of ATG5 increases motor axon regeneration, establishing a connection between ATG5 expression and the process of motor neuron regeneration in the context of neuronal damage.

**Figure 3:**
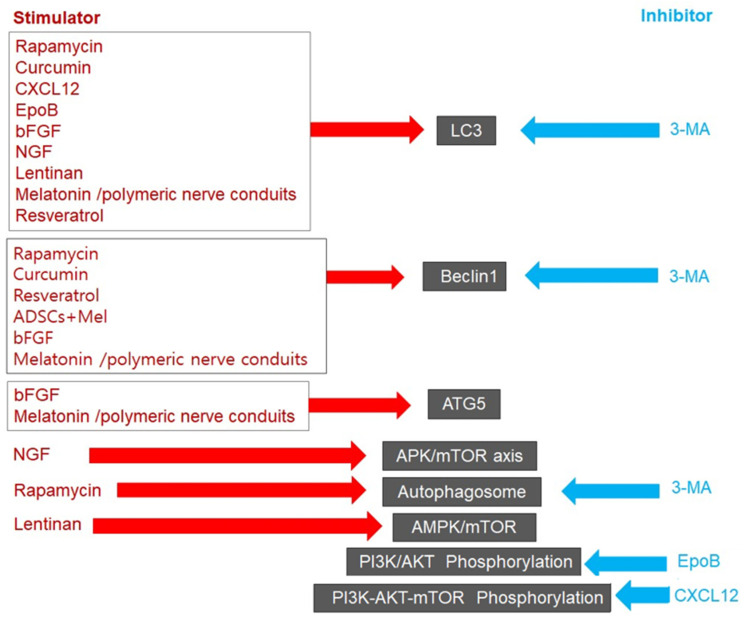
Schematic of the expression of autophagy-related factors in peripheral nerves with or without stimulation of autophagy-inducing/inhibiting factors after peripheral nerve injury.

**Table 1:** Expression of autophagy-related factors in sciatic nerves without stimulation of autophagy-inducing factors after sciatic nerve injury.

Author/Year/Reference	Study Design	Species and/or Sample	Nerve/Injury Methods	Detection Method	Target Substance(s) Associated with Autophagy	Results: Conclusions
Gomez-Sanchez et al., 2015 [19]	Animal study	*Atg7^flfl^* mice, *Mpz*-Cre mice, *Mpz*-Cre^+^, *Atg7*^loxP/loxP^ mice	Sciatic nerve transection	Immunohistochemistry Electron microscopy qRT-PCR Western blotting Lipid analysis Proteomic analysis Behavioral tests	ATG5, ATG7, ATG9, ATG12, LC3-II	Several autophagy-related genes were expressed during demyelination; in particular, the ULK complex, ATG9 cycling system, and ATG7, which are essential for autophagosome formation, were strongly induced. ATG7, ATG5, ATG12, and LC3 II were significantly reduced in homogenates of uninjured and injured nerves from *Atg7*-cKO mice, suggesting impaired autophagy. These results reveal an important role for inductive autophagy during Wallerian degeneration and point to potential mechanistic targets for accelerating myelin clearance and improving demyelinating disease.
Romeo-Guitart et al., 2019 [20]	Animal study	Sprague–Dawley rats	Sciatic nerve/crush and transection injury	Immunohistochemistry Western blotting Cell culture Electrophysiological and functional assessments	Atg5	Nerve conduction amplitudes were higher in gastrocnemius and tibialis anterior muscles in the AAVrh10-ATG5 group than in the AAVrh10-GFP group at 48 days and 60 days post-injury. ATG5 overexpression in spinal motoneurons stimulates mTOR-independent autophagy and facilitates a growth-competent state after nerve transection, improving motor axonal regeneration and electromyographic responses.

AAV, adeno-associated virus; ATG, autophagy-related genes; GFP, green fluorescent protein; LC3-II, microtubule-associated protein-1 light chain 3-II protein; mTOR, mammalian target of the rapamycin; qRT-PCR; quantitative reverse transcription-polymerase chain reaction.

### 3.2. Activation of Autophagy and Expression of Related Factors in Facial and Sciatic Nerves with Stimulation of Autophagy-Inducing Factors after Peripheral Nerve Injury (Table 2) (Figure 3)

#### 3.2.1. Autophagy Responses to Rapamycin and 3-Methyladenine 

The influence of rapamycin, an allosteric inhibitor of mTOR—and thus an activator of autophagy—and 3-methyladenine (3-MA), an autophagy inhibitor, on the response to nerve injury was tested in adult female Sprague–Dawley rats. In these studies [21], the sciatic nerve was clamped with forceps three times for 10 s each at 10-s intervals; for sham controls, the nerve was exposed without performing a crush injury. Five days after surgery, rats were intraperitoneally injected with rapamycin (1 mg/kg; crush + Rapa group), 3-MA (50 mg/kg; crush + 3-MA group), or vehicle (DMSO) control (crush + Veh group). A fourth group received sham surgery and vehicle injection (sham + Veh group). Motor function was assessed by evaluating standing time and footprint intensity of the operated limbs at 1, 2, 3, and 6 weeks after injury. Mean standing time in the rapamycin-treated group was 2-fold longer than that in the crush + Veh group at 1 and 2 weeks after injury (*p* < 0.05), whereas mean footprint strength was similar between the two groups at 2 weeks. These results indicate that animals treated with rapamycin are better able to support body weight on the injured limb from 2 weeks after injury, probably as a result of enhanced autophagy in the acute phase. In contrast, average standing time in the 3-MA group was significantly lower compared with sham-operated animals at 1 and 2 weeks post-injury (45% and 15%, respectively), and mean footprint strength was approximately 20–30% lower (*p* < 0.05). These results provide evidence supporting the role of autophagy in nerve regeneration and motor function recovery in the peripheral nervous system, and may serve as a basis for the development of therapies that improve the outcome of peripheral nerve injury through pharmacological induction of autophagy.

In a related set of experiments [22], sciatic nerves of 8-week-old male Sprague–Dawley rats (n = 42) were dissected instead of clamped and autophagy was studied. In this study, rats were randomly divided into four groups: sham neurorrhaphy (control group), transected + DMSO group (sciatic nerve transected, repaired, and then injected with DMSO), Rapa group (sciatic nerve transected, repaired, and then injected with rapamycin), and a 3-MA group (sciatic nerve transected, repaired, and then injected with 3-MA). The sciatic nerve function index (SFI), which ranges from −10 (normal) to −100 (severe damage), was used as a recovery metric. There was no change in the sham-treated control group, whereas rats injected with rapamycin to induce autophagy showed improved hindlimb function (SFI: −44.56 ± 5.86) compared with rats injected with DMSO (−59.74 ± 5.11; *p* < 0.05). In contrast, functional recovery in rats injected with 3-MA was significantly inhibited (SFI: −72.15 ± 15.84; *p* < 0.05). Immunostaining for S-100 and LC3 increased in the Rapa group; merged staining also increased in the Rapa group (115.5 ± 11.56) compared with DMSO (28.5 ± 4.203 pixels/cm^2^; *p* < 0.001), but decreased in the 3-MA group (10 ± 4.546 pixels per cm^2^; *p* < 0.05). In addition, neurofilament immunostaining and merged staining with LC3 tended to increase in the Rapa group, suggesting improved neurofilament continuity and nerve function. Collectively, these findings reinforce the importance of autophagy in peripheral nerve regeneration and suggest its role in scar reduction.

Another related study [23] focused on the inferior alveolar nerve (IAN) instead of the sciatic nerve. In this study, the IAN of Sprague–Dawley rats was transected, and rapamycin, 3-MA, or vehicle was administered to the IAN transection (IANX) site once a day for 10 days after surgery. All rats that underwent surgery failed to show a motor response in the lower lip for 4 days after IANX. The elevated head-withdrawal threshold (HWT) gradually decreased from day 6 after IANX; in contrast, the head-withdrawal threshold (HWT) of IANX rats was significantly higher than that of control rats throughout the experimental period. In sections proximal and distal to the severed IAN, rapamycin administration to the injured area led to a significant reduction in the expression level of phosphorylated p70S6K (p-p70s6k), a downstream target of mTOR. In contrast, beclin-1 and microtubule-associated protein-1 light chain 3-II (MAP1LC3) protein levels in the proximal and distal stumps of the severed IAN were increased in the Rapa group compared with the control group. Thus, recovery from IANX-induced sensory disturbance of the lower lip was markedly improved by rapamycin. These results suggested that rapamycin administration is a promising treatment for IANX-induced sensory impairment.

#### 3.2.2. Curcumin

Given its intricate interactions with diverse signaling pathways and potential to modulate an array of cellular processes, curcumin—a natural product with broad health benefits and low toxicity—is an attractive candidate for investigating in the context of nerve injury [24]. In one such investigation [25] employing a rat model of sciatic nerve injury, the sciatic nerve of male Sprague–Dawley rats was clamped with a 2-mm-wide needle holder, after which nerve-damaged rats were randomly divided into four groups (n = 10 rats/group): negative control (NC), curcumin, PD98059 (MEK inhibitor) + curcumin, and insulin-like growth factor 1 (IGF 1) + curcumin. In groups treated with curcumin, rats were intraperitoneally administered curcumin (100 mg/kg/d, dissolved in DMSO at 20 mg/mL) for 60 days. Following surgery, LC3-II and beclin-1 levels significantly increased, whereas the levels of p62 significantly decreased in all curcumin-containing groups compared with the NC group, suggesting initiation of autophagy in the damaged sciatic nerve. The greatest increase in LC3-II and beclin-1 was detected in the group given curcumin alone, suggesting that injury-induced autophagy in the sciatic nerve is enhanced by curcumin treatment. Curcumin administration also significantly increased the levels of phosphorylated-ERK1/2 (p-ERK1/2) and decreased levels of p-Akt, indicating that curcumin promotes ERK1/2 pathway activation and inhibits Akt/mTOR pathway activation in the injured sciatic nerve. Taken together, these observations indicate that curcumin promotes injury-induced cell autophagy in the rat sciatic nerve by targeting Akt/mTOR and ERK1/2 pathways.

#### 3.2.3. Three-Dimensional Melatonin Nerve Scaffolds

Damage to peripheral nerves gives rise to various disorders in patients. In the case of nerve transection, autogenous nerve transplantation is a good standard treatment for severe peripheral nerve defects. However, a number of factors, including post-traumatic inflammation, oxidative stress, and misdirected nerve regeneration, can inhibit functional recovery of peripheral nerves. One widely used and effective method for bolstering peripheral nerve regeneration is to position a nerve conduit between two transected nerves to support nerve regrowth. Although this method is not sufficient for long nerve defects, it is effective for short defects, with traditional polymeric nerve conduits such as polyglycolic acid and polycaprolactone (PCL) seeing wide use in clinical practice [26,27]. In one preclinical study [28], 30 male Sprague–Dawley rats (weight, 200–250 g) were randomly assigned to three groups: Mel (melatonin)/PCL, PCL, and autologous transplant groups. After the sciatic nerve segment was cut, the two nerve endings were ligated using a 15-mm nerve guide conduit. SFI, used as a metric to assess recovery, was significantly higher in the Mel/PCL group than the PCL group at both 6 and 12 weeks postoperatively (*p* < 0.05). At 6 weeks, SFI remained lower in Mel/PCL than in the autograft group (*p* < 0.05), showing good results for nerve regeneration and nerve recovery. The function and role of autophagy in the regenerated sciatic nerve was confirmed by assessing LC3A/B expression by immunohistochemical analysis. Beclin-1 and LC3-I expression were 2.6- and 4.1-fold higher, respectively, in the Mel/PCL group than in the PCL group (*p* < 0.05). ATG3, ATG5, and ATG7 expression were also higher in the Mel/PCL group than the PCL group, although this increase was more modest (1.5-, 1.8-, and 2.2-fold, respectively). These results show that the Mel/PCL conduit can clear nerve debris and trigger autophagy, fostering nerve regeneration.

#### 3.2.4. CXCL12 

CXCL12 has garnered interest as a potential therapeutic target for enhancing nerve regeneration, and emerging evidence suggests that it might play a role in regulating autophagy. In one study investigating this connection [29], male Sprague–Dawley rats were separated into surgery and sham groups. Rats in the surgery group were subjected to nerve-crush injury of the main trunk of the right extracranial facial nerve (compressed once using mosquito forceps for 50 s at a point 0.5 cm from the stylomastoid foramen), with and without treatment with recombinant rat CXCL12, injected near the posterior mastoid of the rat ear at a dose of 4 μg/kg/d. The CXCL12 group was further subdivided into 3- and 28-day subgroups. Rats were sacrificed on days 0, 1, 3, 7, 17, and 28, and CXCL12 levels and expression of various markers of autophagy were assessed at each time point. 

Expression of endogenous CXCL12 protein was largely unchanged in sham-operated rats. However, in the nerve-injured experimental group, CXCL12 expression transiently increased 1 day after FNI, decreased at 14 days, and returned to normal by 28 days. These dynamic changes in the expression of CXCL12 increasing after FNI and later decreasing suggest that CXCL12 may be involved in repair of FNI. Administration of CXCL12 after FNI increased levels of LC3B II and decreased p62, two markers of autophagy, in a time-dependent manner in Schwann cells, producing a maximum effect at 24 h. Treatment with CXCL12 also markedly reduced the expression of p-PI3K, p-AKT, p-mTOR, and p-S6, and significantly increased the number of autophagosomes after 24 h compared with the control group, suggesting that CXCL12 increases autophagy in Schwann cells. Taken together, these observations demonstrate that CXCL12 exerts a therapeutic effect on FNI. The fact that CXCL12 plays a pivotal role in the regulation of Schwann cell migration through enhancement of autophagy by the PI3K-AKT-mTOR signaling pathway further reinforces the possible importance of CXCL12 in FNI.

#### 3.2.5. Resveratrol 

Resveratrol, which has been investigated for potential therapeutic interventions by virtue of its multifarious effects, has been shown to modulate autophagy by influencing the key autophagy-regulating signaling pathways, mTOR and AMPK [30]. In one study investigating the potential of resveratrol to promote recovery after nerve injury [31], resveratrol (100 mg/kg/d) was administered intraperitoneally for 7 days to rats subjected to sciatic nerve crush injury (SNCI). In addition to the resveratrol treatment group (crush + Res) were a 3-MA treatment group (crush + 3-MA), a vehicle control group (crush + Veh), and a sham-operated group. SFI, measured as an index of functional recovery, was determined for each rat on days 7 and 14 after SNCI. SFI was significantly higher in the crush + Res group on days 7 (*p* < 0.01) and 14 (*p* < 0.05) after nerve injury compared with the control group. On day 28, the SFI for the crush + Res group was higher than that for the crush + Veh and crush + 3-MA groups (*p* < 0.05). The SFI for the crush + 3-MA group was lower than that for the crush + Veh group (*p* < 0.01). Levels of the myelin-specific protein, MPZ, and autophagy-related proteins, LC3B, p62, and beclin-1, in sciatic nerve slices were confirmed by Western blotting. On day 4 post-SNCI, the expression of MPZ in the distal end of the injured nerve was significantly lower than that in the control group. Levels of LC3-II/I and beclin-1 were highest in the crush + Res group and lowest in the sham group. Collectively, these results show that resveratrol can promote the recovery of damaged peripheral nerves, consistent with previous reports that resveratrol improves autophagy in Schwann cells, helps peripheral nerve repair, and partially accelerates the process of Wallerian degeneration after SNCI.

#### 3.2.6. Nerve Growth Factor 

Nerve growth factor (NGF) has an established role in nerve development and repair and is known to modulate the AMPK/mTOR pathway; as such, it has the potential to modulate autophagy and contribute to nerve damage and recovery mechanisms. The role of NGF in modulating autophagy was investigated [32] in a rat sciatic nerve crush-injury model in which 8-week-old male Wistar rats subjected to nerve injury were administered 0.2 mL of an NGF solution (20 μg/mL), designated the peripheral nerve injury plus NGF (PNI + NGF) group, or the same amount of saline (PNI group), injected intramuscularly once a day for 5 days. NGF treatment (PNI + NGF group) increased the fluorescence intensity of the autophagy marker protein, LC3, compared with saline (PNI group). The regulation of NGF-mediated autophagy in Schwann cells during Wallerian degeneration by p75NTR/AMPK/mTOR signaling was confirmed by examining the levels of p-AMPK, p-p70s6k, and p-mTOR in the sciatic nerve of injured mice by Western blotting 5 days after PNI. Compared with results obtained in the control group, the p-AMPK/AMPK ratio increased after PNI whereas p-mTOR/mTOR and p-p70s6k/p70s6k ratios decreased after PNI. p75NTR is highly expressed in Schwann cells [33], and NGF stimulates downstream signaling pathways by activating p75NTR [34]. AMPK/mTOR signaling is also known to exert neuroprotective effects against focal cerebral ischemia through activation of autophagy [35], and AMPK upregulates autophagy by inhibiting mTOR activation [36]. Collectively, these studies show that administration of NGF not only activates autophagy in dedifferentiated Schwann cells and accelerates myelin debris removal and phagocytosis, but it also promotes axon and myelin regeneration in the early stage after PNI.

#### 3.2.7. Lentinan 

Lentinan, a natural product with broad pharmacological activity [37], can modulate the autophagy process by which Schwann cells remove myelin fragments after PNI. The potential to harness this activity to promote recovery after nerve injury was investigated in rats subjected to sciatic nerve compression injury (SNCI) [38]. After injury, the experimental group received 20 mg/kg of lentinan by intraperitoneal injection every day for 14 days, and the control group was injected with normal saline, after which neurological recovery was evaluated in each group. Interestingly, although both groups showed improvement in sciatic nerve function over time, a difference between the control group and the lentinan group appeared after 7 days of treatment and became more pronounced after 2 weeks, at which point the SFI for the lentinan group was significantly higher than that for the PNI (saline-treated) group. In addition, the lentinan group showed delayed muscle atrophy and a much higher muscle wet-weight ratio than the PNI group. These results suggest that lentinan can significantly promote early functional recovery after SCNI. Lentinan promoted neurological recovery by stimulating autophagic flux in vivo through the AMPK/mTOR signaling pathway, accelerating the removal of myelin debris by Schwann cells and inhibiting neuronal cell death. It was further found that the LC3-II/LC3-I ratio, an important marker of autophagic flux that reflects the involvement of LC3 in the formation of autophagosomes during autophagy induction, increased in the PNI group after nerve compression injury. Lentinan also promoted phagocytosis of myelin fragments by Schwann cells in vitro. Collectively, these results suggest that lentinan accelerates the autophagic clearance of myelin debris in Schwann cells, a process likely regulated by the AMPK/mTOR signaling pathway that mainly promotes nerve regeneration.

#### 3.2.8. Adipose-Derived Stem Cells 

Adipose-derived stem cells (ADSCs) have the capacity to bolster nerve regeneration through modulation of autophagy activity [39]. The contribution of ADSCs in harnessing autophagy-related mechanisms for effective nerve repair was investigated [40] in a rat PNI model in which sciatic nerves of Sprague–Dawley rats (n = 12) were crushed with hemostatic forceps (three times for 10 s each at 10-s intervals) and then divided into the following groups: Control group (sham + PBS), Model group (crush + PBS), ADSCs + Mel group (crush + ADSCs + melatonin), and 3-MA group (crush + 3-MA). Compared with the control group, the SFI for the Model group decreased 21.1-fold (*p* < 0.01), indicating limb dysfunction after nerve injury. SFI values exhibited time-dependent increases in treatment group rats, measured 1, 2, 3, and 4 weeks after surgery, indicating improved recovery of neurological function in treated rats compared with crush + PBS rats (*p* < 0.01). Recovery of regenerated nerves 4 weeks after treatment, based on SFI values, was best for the ADSCs + Mel group compared with Mel and ADSC groups (*p* < 0.01). The ADSCs + Mel group showed significantly increased myelin regeneration and number of motor neurons as well as reduced gastrocnemius atrophy. An analysis performed 1 week after PNI showed a significant increase in the number of autophagosomes and lysosomes and elevated expression of LC3-II/LC3-I and beclin-1 protein in the ADSCs + Mel group compared with the Model group; by comparison, these indicators were significantly decreased in the 3-MA group. After 4 weeks, sciatic nerve function in the ADSCs + Mel group was similar to that of the Control group. A quantitative analysis revealed an increase in the LC3-II/LC3-I ratio and protein levels of beclin-1 in the Model group compared with the control group (*p* < 0.01). Beclin-1 levels were also increased in ADSCs, Mel, and ADSCs + Mel groups compared with controls (*p* < 0.01). In addition, the LC3-II/LC3-I ratio and beclin-1 protein levels were higher in the ADSCs + Mel group (*p* < 0.01) compared with ADSC- and Mel-only groups. The effects of 3-MA on the LC3-II/LC3-I ratio and beclin-1 protein levels were opposite to those on the ADSCs + Mel group (*p* < 0.01). This study showed that various indices of nerve regeneration were significantly reduced after treatment with the autophagy inhibitor 3-MA. Taken together with the improved outcome observed in the ADSCs + Mel group, these observations suggest that ADSCs combined with Mel can promote sciatic nerve regeneration in rats by altering autophagy activity in the early stages of sciatic nerve injury.

#### 3.2.9. Basic Fibroblast Growth Factor 

Although studies have shown that basic fibroblast growth factor (bFGF) can activate autophagy and promote peripheral nerve repair, the role of bFGF in the facial nerve and its molecular mechanism of action are not clear. In a study designed to address this question [41], male Sprague–Dawley (SD) rats were randomly divided into five groups (n = 10/group): sham group, FNI group (FNI + saline), poloxamer group (FNI + poloxamer), bFGF group (FNI + bFGF), and P-bFGF group (FNI + P-bFGF). The FNI model was created by clamping the facial nerve for 60 s with hemostatic forceps. Normal saline (control) or bFGF (0.1 mg/mL) was administered on days 0, 3, and 7, and rats were sacrificed on day 7. Facial nerve morphological abnormalities induced by FNI were improved in bFGF and P-bFGF groups compared with the FNI group. Nerve fibers in the P-bFGF group displayed significantly more regular regeneration than those in the bFGF group. The fluorescence intensity of LC3B was slightly increased in the FNI group, but was enhanced to a greater degree in bFGF and P-bFGF treatment groups, with the P-bFGF group showing significantly greater LC3B intensity than the bFGF group. Western blot analyses showed that LC3B-II, beclin-1, and ATG5 expression paralleled changes in the fluorescence intensity of LC3B. Taken together, the results of this study showed that P-bFGF effectively promotes cell proliferation, myelination and functional recovery, and reduces neuronal apoptosis after FNI.

#### 3.2.10. Facilitation by Epothilone B 

Epothilone B (EpoB) is an FDA-approved antineoplastic agent known for its ability to induce α-tubulin polymerization and improve microtubule stability [42]. It has also recently gained attention for its regenerative effects on the central nervous system. However, the potential therapeutic effects of EpoB on peripheral nerve regeneration has only just begun to be investigated. In one such study [43], a sciatic nerve injury model was created by compressing the sciatic nerve of adult male Sprague–Dawley rats with a vascular clamp for 50 s. Rats in the EpoB group (n = 10 rats/group) received an intraperitoneal injection of 1 mL of 150 µg/mL EpoB every day for 7 days. SFI values were not significantly different between control and EpoB groups within the first 2 weeks. However, the SFI of the EpoB group was significantly increased at both 3 weeks (−27.50 ± 1.58) and 4 weeks (−17.32 ± 2.17) compared with the control group, where the 4-week value was −29.73 ± 1.97 (*p* < 0.05 at both time points), suggesting that EpoB treatment achieved superior recovery of motor function. At 24 h after EpoB treatment, LC3B II levels were significantly increased and PI3K and Akt phosphorylation were significantly inhibited in Schwann cells (*p* < 0.05). Transmission electron microscopy further showed that EpoB significantly increased the number of autophagosomes (5 ± 1) in Schwann cells at this time point compared with controls (2 ± 1; *p* < 0.05). To further investigate the role of autophagy in EpoB-enhanced SC migration, this study also tested the effect of 3-MA on cell migration, demonstrating that this autophagy inhibitor significantly inhibited EpoB-induced autophagosome production in Schwann cells (*p* < 0.05) and abolished EpoB-enhanced migration of these cells (*p* < 0.05). Collectively, these results suggest that EpoB induces autophagy and that this autophagy is involved in EpoB-enhanced migration of Schwann cells.

**Table 2:** Expression of autophagy-related factors in facial and sciatic nerves with stimulation of autophagy-inducing/inhibiting factors after peripheral nerve injury.

Author/Year/Reference	Study Design	Species and/or Sample	Nerve/Injury Methods	Autophagy stimulants/Inhibitors Detection Method	Target Substances Associated with Autophagy	Results: Conclusions
Huan et al., 2016 [21]	Animal study	Sprague–Dawley rats	Sciatic nerve crush injury	Rapamycin and 3-MA Electron microscopy Western blotting Immunocytochemistry CatWalk gait analysis	Autophagosome LC3-II	The mean standing time in the Rapa group was two-fold higher than in the crush + Veh group at 1 and 2 weeks post-injury (*p* < 0.05). In contrast, the mean standing time was significantly lower in the 3-MA group at 1 and 2 weeks post-injury (45% and 15%, respectively), whereas the mean footprint intensity was ~20–30% lower at 1, 2, and 3 weeks post-injury compared with sham-operated animals (*p* < 0.05). Expression of the autophagy marker LC3-II was higher in the Rapa group and lower in the 3-MA group (*p* < 0.05) compared with controls. Modulation of autophagy in PNI could be an effective pharmacological approach for promoting nerve regeneration and reestablishing motor function.
Ko et al., 2017 [22]	Animal study	Sprague–Dawley rats	Sciatic nerve transection	Rapamycin or 3-MA Behavioral test Immunohistochemistry	LC3	Induction of autophagy by injection (i.p.) of rats with rapamycin significantly improved hindlimb function compared with injection of DMSO (−44.56 ± 5.86 vs. −59.74 ± 5.11; *p* < 0.05). Neurofilament, LC3, and merged staining trended higher in the Rapa group. A deficiency of Schwann cell autophagic activity might be an early event in nerve scar formation, and modulating autophagy might be a powerful pharmacological approach for improving functional outcomes.
Zhao et al., 2017 [25]	Animal study	Sprague–Dawley rats	Sciatic nerve compression (mechanical clamping)	Curcumin Western blotting qRT-PCR Flow cytometry	LC3-II Beclin-1	Curcumin treatment increased the levels of LC3-II and beclin-1 in the negative control group (*p* < 0.05). Curcumin accelerated repair of injured sciatic nerves in rats by reducing Schwann cell apoptosis and promoting myelinization.
Yun Qian et al. 2018 [28]	Animal study	Sprague–Dawley rats	Sciatic nerve dissection	Melatonin and polymeric nerve conduits Walking tract analysis Morphological evaluation Immunofluorescence evaluation Western blotting	Beclin-1 LC3A/B Atg3 Atg5 Atg7	LC3A/B expression was significantly higher in MLT/PCL and autograft groups than the PCL group (*p* < 0.05). Beclin-1 and LC3-I expression were 2.6- and 4.1-fold higher, respectively, in the MLT/PCL group than in the PCL group (*p* < 0.05). ATG3, ATG5, and ATG7 expression were 1.5-, 1.8-, and 2.2- fold higher in the MLT/PCL group than the PCL group, but were significantly lower in both groups compared with the autograft group (*p* < 0.05). 3D melatonin nerve scaffolds increase autophagy in peripheral nerve regeneration.
Gao et al., 2019 [29]	In vivo	Sprague–Dawley rats	Facial nerve compression injury (mechanical clamping)	CXCL12 Cell proliferation assay TUNEL staining ELISA Immunofluorescence Western blotting TEM	LC3II/I PI3K-Akt-mTOR	CXCL12 induced a time-dependent increase in LC3B II and reduction in P62—two markers of autophagy—in Schwann cells. Treatment with CXCL12 decreased PI3K, AKT, and mTOR phosphorylation, but increased expression of the autophagy marker LC3II/I. CXCL12 promotes the migration of Schwann cells and is potentially a key molecule in the repair of FNI.
Zhang et al. 2020 [31]	Animal study	Sprague–Dawley rats	Sciatic nerve crush injury	Resveratrol SFI TEM Immunohistochemistry Western blot analysis	LC3-II/IBeclin1	SFI values on days 7 and 14 post-injury were significantly higher in the crush + Res group than the control group (*p* < 0.01). Levels of LC3-II/I and beclin-1 were highest in the crush + Res group and lowest in the sham group. Resveratrol facilitated peripheral nerve repair by improving autophagy in Schwann cells, at least partially accelerating the Wallerian degeneration process after SNCI.
Li et al., 2020 [32]	Animal study	Wistar rats	Sciatic nerve crush injury	NGF Electron microscopy Immunofluorescence Western blotting qRT-PCR Myelin phagocytosis assays Detection of autophagic flow	LC3 p75NTR/AMPK/mTOR axis	NGF treatment resulted in an increase in the fluorescence intensity of the autophagic marker protein LC3 in the PNI + NGF group compared with the PNI group. Compared with the sham group, the p-AMPK/AMPK ratio increased whereas both p-mTOR/mTOR and p-p70s6k/p70s6k ratios decreased after PNI. These effects were further enhanced by NGF treatment in the PNI + NGF group. The effect of NGF on promoting early nerve regeneration is closely associated with its acceleration of autophagic clearance of myelin debris in Schwann cells.
Inada et al., 2021 [22]	Animal study	Sprague–Dawley rats	Inferior alveolar nerve transection	Rapamycin 3-MA Western blotting Immunohistochemistry Mechanical sensitivity assay	Beclin-1 LC3	The head-withdrawal threshold (HWT) in IANX rats was significantly higher than that in sham-operated rats throughout the experimental period. Rapamycin increased both LC3-II and beclin-1 expression in the injured site of the inferior alveolar nerve on day 6 after nerve transection. Rapamycin-induced facilitation of recovery from the sensory disturbance following IANX was mediated through the clearance of myelin debris by Schwann cells.
Xiao et al., 2022 [38]	Animal study	Sprague–Dawley rats	Sciatic nerve compression injury	Lentinan Gait analysis Network pharmacology Histological analysis Immunofluorescence Cell culture Western blotting	AMPK/mTOR LC3-II/LC3-I ratio	Lentinan promoted autophagic flux in vivo via the AMPK/mTOR signaling pathway, accelerated the clearance of myelin debris by SCs, and inhibited neuronal apoptosis, thereby promoting neurological recovery. Lentinan treatment increased the LC3-II/LC3-I ratio. Lentinan promotes nerve regeneration primarily by accelerating the autophagic clearance of myelin debris in Schwann cells, a process likely regulated by the AMPK/mTOR signaling pathway.
Zhang et al., 2022 [40]	Animal study	Sprague–Dawley rats	Sciatic nerve crush injury	ADSCs + Mel SFI Western blotting Immunofluorescence	LC3-II/LC3-I Beclin-1	The number of autophagosomes and lysosomes and the expression of LC3-II/LC3-I and beclin-1 proteins were prominently increased in the ADSCs + Mel group compared with the Model group, and significantly decreased in the 3-MA group. ADSCs combined with melatonin promotes sciatic nerve regeneration in rats by altering the early autophagic activity of the injured sciatic nerve.
Hu et al., 2022 [41]		Sprague–Dawley rats	Facial nerve compression injury (mechanical clamping)	bFGF Fluorescence labeling and imaging Western blotting Facial nerve functional scoring	LC3B Beclin-1 ATG5	Abnormal nerve morphologies were improved in the bFGF group and P-bFGF group. Nerve fibers in the P-bFGF group showed significantly greater and more regular regeneration compared with those in the bFGF group. Immunofluorescence staining showed that the fluorescence intensity of LC3B was slightly increased by FNI and was further enhanced by bFGF or P-bFGF treatment, with P-bFGF treatment producing significantly greater fluorescence intensity than bFGF. Western blotting showed that changes in the expression of LC3B-II, beclin-1, and ATG5 paralleled changes in the fluorescence intensity of LC3B. P-bFGF effectively promotes cell proliferation, myelination, and functional recovery, and also reduces apoptosis of nerve cells after FNI.
Zhou et al., 2020 [43]	Animal study	Sprague–Dawley rats	Sciatic nerve crush injury	EpoB Walking track Assessment Western blotting Von Frey filament test TEM	PI3K/Akt	SFI values for EpoB-treatment groups gradually decreased over 4 weeks, indicating that EpoB treatment facilitated the recovery of sensory function after nerve crush injury. EpoB treatment significantly increased LC3B II levels and inhibited PI3K and Akt phosphorylation in Schwann cells. EpoB is capable of promoting axonal regeneration and remyelination as well as functional recovery in a rat model of nerve crush injury.

ADSC, adipose-derived stem cells; AMPK, activated protein kinase; Akt, protein kinase B; ATG, autophagy-related genes; bFGF, basic fibroblast growth factor; ELISA, enzyme-linked immunoassay; EpoB, epothilone B; IANX, inferior alveolar nerve transection; LC3-II, microtubule-associated protein-1 light chain 3-II protein; Mel, melatonin; 3-MA, 3-methyladenine; mTOR, mammalian target of the rapamycin; MLT/PCL, melatonin and polymeric nerve conduits; NGF, nerve growth factor; PNI, peripheral nerve injury; p75NTR, 75 kD neurotrophin receptor; PI3K; phosphatidylinositol 3-kinase; qRT-PCR; quantitative reverse transcription-polymerase chain reaction; SNCI, sciatic nerve compression injury; SFI, sciatic nerve function index; TEM, transmission electron microscopy.

## 4. Conclusions

Although research on the facial nerve remains limited, studies of autophagy conducted on peripheral nerves, including the sciatic nerve, have yielded noteworthy findings. These investigations have revealed the expression of a number of autophagy-related substances in compression and transection nerve injury models, including AMPK, Atg3, Atg5, Atg7, Atg9, Atg12, beclin-1, LC3, LC3 II/I, mTOR, PI3K, PI3K/Atk, PI3K-Akt-mTOR, and p75NTR/AMPK/mTOR, as well as changes in autophagosome dynamics. In the case of PNI, administration of autophagy inducers or inhibitors increased or decreased autophagy-related substances, suggesting that autophagy is closely related to peripheral nerve degeneration and regeneration. It is imperative that future studies delve in other unexplored autophagy-related factors post-nerve damage and further explore the intricate interplay between autophagy, inflammation- and immunity-related factors, and nerve regeneration-related factors.

## Data Availability

Not applicable.
